# Arthroscopic versus open, medial approach, surgical reduction for developmental dysplasia of the hip in patients under 18 months of age

**DOI:** 10.1080/17453674.2019.1599775

**Published:** 2019-04-02

**Authors:** Serda Duman, Yalkin Camurcu, Hakan Sofu, Hanifi Ucpunar, Deniz Akbulut, Timur Yildirim

**Affiliations:** a Diyarbakir Selahaddin Eyyubi State Hospital, Department of Orthopaedics and Traumatology, Diyarbakir;;; b Erzincan University Faculty of Medicine, Department of Orthopaedics and Traumatology, Erzincan;;; c Medical Park Bahçelievler Hospital, Department of Orthopaedics and Traumatology, Istanbul;;; d Baltalimani Bone and Joint Diseases Education and Research Hospital, Department of Pediatric Orthopaedics, Istanbul

## Abstract

Background and purpose — The value of arthroscopic surgical reduction in developmental hip dysplasia is poorly known. We compared the clinical and radiographic efficacy of arthroscopic and medial open surgical reduction in patients less than 18 months of age with developmental hip dysplasia.

Patients and methods — 54 patients with a mean age of 11 months who were treated by Ludloff’s medial open reduction technique (28 hips, Group L) or arthroscopic surgical reduction technique (26 hips, Group A) were evaluated in this case series. Data on age, sex, preoperative Tönnis grade, operative time, estimated blood loss, residual leg length discrepancy, range of motion (ROM), acetabular index (AI) angle, coverage ratio of the femoral head, continuity of Menard–Shenton line, re-dislocation rate, McKay classification, and Kalamchi–MacEwen avascular necrosis (AVN) classification were collected.

Results — Preoperatively, the mean AI angle was 39° in Group L and 37° in Group A. At the latest follow-up, the mean AI was 26° in both groups. The mean femoral head coverage ratio was 79% in Group L and 80% in Group A. The Menard–Shenton line was intact in all patients. Residual leg length discrepancy or limited ROM was not detected in any patients. 4 patients in Group L and 2 in Group A were diagnosed with type 2 AVN.

Interpretation — Arthroscopic surgical reduction in patients aged 6–18 months revealed promising clinical and radiographic outcomes similar to medial open reduction using Ludloff’s technique.

Arthroscopic surgical reduction in developmental dysplasia of the hip (DDH) is an innovative technique (Bulut et al. 2005, Öztürk et al. 2013, Eberhardt et al. 2014). Iliopsoas tenotomy, capsular release, and excision of the ligamentum teres, as well as pulvinar tissue, can easily be performed arthroscopically (Eberhardt et al. 2015), preferably in children aged younger than 18 months. Previous reports in the literature have focused on the detailed surgical technique and outcomes of this approach (Eberhardt et al. 2012, 2015, Öztürk et al. 2013).

We compared the clinical and radiographic efficacy of arthroscopic and open surgical reduction in children aged younger than 18 months with DDH.

## Patients and methods

**Figure 1.**
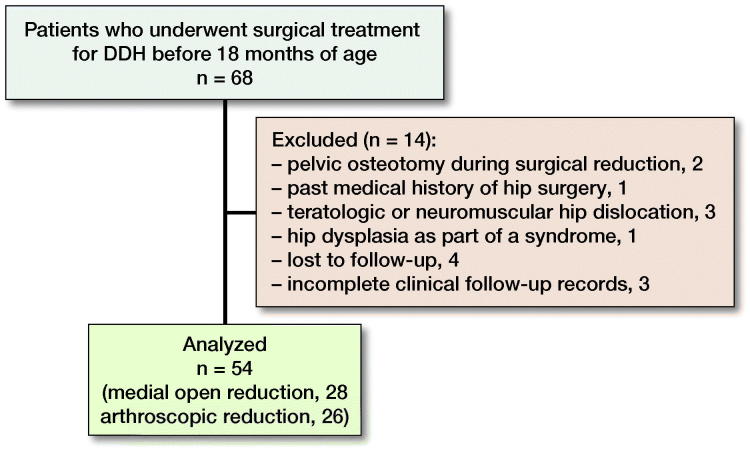
Flow chart demonstrating the excluded patients.

This case series evaluated the clinical and radiographic data of patients who underwent surgical treatment for DDH. The inclusion criteria were primary DDH treated surgically using a medial open or an arthroscopic approach in patients aged 6–18 months with a minimum postoperative follow-up of 24 months. Of 68 patients we excluded 14 for different reasons (Figure 1).

**Figure 2.**
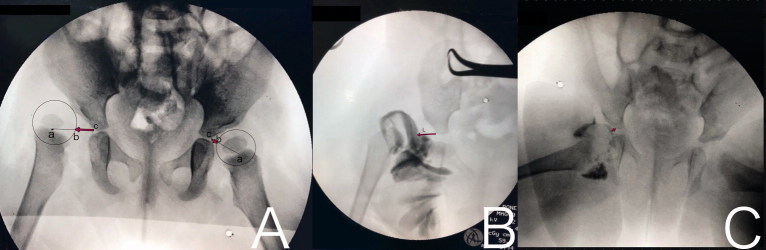
A. L distance (red arrow). The distance between the iliac bone and the surface of the cartilaginous femoral head (b) on a line drawn from the inferior end of the ilium (c) to the center of the cartilaginous femoral head (a). B. Arthrogram of a dislocated hip with elongated L distance. C. Arthrogram of a hip with an acceptable reduction.

Arthrography was performed in all patients under 1 year of age, and we decided to perform surgery when concentric reduction could not be achieved. The accepted criterion for adequate reduction of the hip was a concentrically reduced cartilaginous femoral head with < 4 mm of lateralization (L distance). Lateralization was measured on arthrograms as the distance between the iliac bone and the surface of the cartilaginous femoral head on a line drawn from the inferior end of the ilium to the center of the cartilaginous femoral head, designated as distance L (Figure 2). More than 4 mm of medial dye pooling, a thickened limbus that in some way interferes with reduction, and constricted capsule indicated lack of concentric reduction (Leveuf 1947). Patients older than 1 year underwent surgery (open or arthroscopically) without closed reduction. Additionally, we used the clinical criterion of Ramsey’s safe zone, described as the arc of motion through which the hip remains reduced without forced abduction. To determine the safe zone of Ramsey, the hip was adducted to the point of re-dislocation, and that position was noted. Then, the hip was again reduced and extended until it dislocated, and again point of dislocation was noted as well as the required internal rotation to maintain reduction. The minimum range for the safe zone should be measured as 25°. In the case where the stable zone exceeded the safe zone, excessive forced abduction was needed to obtain concentric reduction, or the safe zone was measured as less than 25°, surgery was indicated. These explicit criteria were consistent between 2 senior pediatric orthopedic surgeons who received training in the same department.

During the study period (2014–2016), 214 patients aged 6–18 months who were diagnosed with DDH underwent treatment at our hospital. Among these, 146 (68%) underwent closed reduction and 68 children (32%) underwent surgery.

After exclusions this study included 47 female and 7 male patients. The mean age of the patients was 11 months (6–17). All patients were diagnosed with unilateral hip joint involvement. For this study the patients were categorized into 2 groups based on the surgical technique used by the 2 authors (1 author routinely performed medial approach open reduction and the other routinely performed arthroscopic reduction). Medial approach open reduction based on the Ludloff technique (Ludloff 1908) was performed in 28 hips (Group L), and arthroscopic reduction was performed in 26 hips (Group A). 14 patients in Group L and 7 patients in Group A received neonatal splinting prior to study enrollment. 2 patients in Group A had failed closed reduction at another center. Age at the time of surgery, sex, the Tönnis grade (Tönnis 1976), and the AI angle measured on anteroposterior pelvic radiographs were recorded in each patient preoperatively.

## Surgical technique

Medial approach open reduction was performed through a transverse incision measuring 5 cm in length. After dissecting the adductor longus muscle, broad exposure of the surgical field was achieved with dissection of the pectineus muscle. Tenotomy of the iliopsoas was performed, and the joint capsule was incised. Subsequently, we excised the ligamentum teres, as well as the pulvinar tissue, followed by incision of the transverse acetabular ligament. The femoral head was then reduced into the acetabular cavity. Post-reduction stability was confirmed, and the surgical incision was sutured. A pelvipedal cast was applied in the human position of 100° flexion, < 50° of abduction, and < 10° of internal rotation.

**Figure 3.**
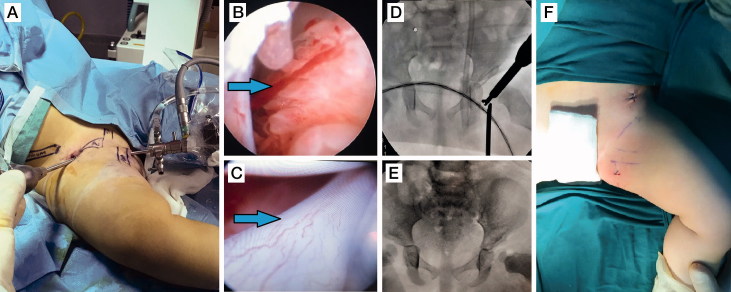
Arthroscopic technique demonstrating the portals (A), intra-articular pulvinar (B), hypertrophic ligamentum teres (C), intraoperative fluoroscopic image during incision of the transverse acetabular ligament (D), and intraoperative radiographic control for concentric reduction (E).

Arthroscopic reduction was performed under general anesthesia with the patient placed in the supine position (Figure 3). An assistant ensured that the hip was maintained in a position of 90° flexion and 40–60° of abduction, and no traction was needed. A medial sub-adductor portal located 1 cm lateral and 1 cm ventral to the ischial tuberosity in the palpable gap between the adductors and the ischiocrural muscles was used to place the 2.7 mm 70° arthroscope intra-articularly under fluoroscopic control (Eberhardt et al. 2015). Arthroscopic evaluation of the hip joint was performed. An anterolateral portal was placed 2 cm distal to the superior iliac spine and 1 cm lateral from a line drawn through the superior iliac spine and the middle of the patella. Radiofrequency ablation and a shaver were used to remove the ligamentum teres and the pulvinar tissue. Arthroscopic scissors were used to incise the transverse acetabular ligament. After intraoperative examination, capsular release with radiofrequency ablation was performed in 5 patients in whom the safe zone was inadequate. Iliopsoas tenotomy was not performed owing to the risks reported by a previous study (Eberhardt et al. 2015). Reduction of the hip joint was then performed, and post-reduction stability was confirmed. Portal incisions were sutured, and a pelvipedal cast was applied in the human position of 100° flexion, < 50° of abduction, and < 10° of internal rotation.

Operative time (min) and the estimated blood loss (mL) were recorded in each patient. Postoperative radiography was performed in all patients to confirm concentric reduction of the femoral head into the acetabular cavity. The pelvipedal cast was removed at the end of the 3rd postoperative month, and a Denis Browne orthosis was applied for an additional 8 weeks with the hip maintained in 30–45° of abduction.

All children were followed-up clinically for at least 24 months (24–30) postoperatively, Residual leg length discrepancy, range of motion (ROM) of the operated hip joint, and changes in the AI (between pre- and postoperative examinations) were assessed in all children. The continuity of the Menard–Shenton line was assessed postoperatively. Coverage ratio of the femoral head inside the acetabulum was also measured (Heyman et al. 1950). The McKay classification (Berkeley et al. 1984) was used to categorize the clinical outcomes in each child. The Kalamchi–MacEwen classification (Kalamichi and MacEwen 1980) was used to evaluate any postoperative avascular necrosis (AVN). All radiographic and clinical measurements or classifications were evaluated and recorded at least twice by 2 authors (YC, DA) who were blinded to the surgical technique.

## Statistics

Owing to the descriptive nature of the analysis used in this case series, descriptive statistics were presented using medians with ranges (minimum–maximum). Frequencies and percentages were calculated to express categorical variables.

## Ethics, funding, and potential conflicts of interest

This study was performed following approval from the institutional ethical review board (33216249-604.01.02-E.37840). Informed consent was obtained from the parents of all children included in this study. No funding was received for this study. All authors declare that they have no conflicts of interest.

## Results (Tables 1 and 2)

The mean operative time was similar in the two groups. However, the mean estimated blood loss in Group A was lower than that in Group L. (No vascular or nerve injury occurred in any patient.) The Menard–Shenton line was intact in all patients postoperatively. On the basis of the McKay classification for functional evaluation of hips, the children were categorized as follows: 18 patients as Grade I, 9 as Grade II, and 1 patient as Grade III in Group L and 16 patients as Grade I and 6 as Grade II in Group A.

Residual leg length discrepancy or limited ROM of the operated hip joint was not detected in any patient during follow-up. Type 2 AVN (Kalamchi–MacEwen AVN classification) was diagnosed in 4 patients in Group L and 2 patients in Group A. Re-dislocation occurred in 1 patient in Group 1 at the end of 1 month postoperatively.

## Discussion

Simple closed reduction is the first-line treatment for DDH in patients aged 6 months to 1 year (Race and Herring 1983, Terjesen and Halvorsen 2007). Open reduction should be considered only if closed reduction cannot be performed. Medial open surgical reduction is a choice for the management of patients younger than 18 months with DDH. The minimal incision and minimal blood loss are advantages of this approach. Limited exposure of the hip joint is a disadvantage. Several studies have described successful outcomes with this technique (Morcuende et al. 1997, Okano et al. 2009). However, injury to the medial circumflex femoral artery with consequent avascular necrosis (AVN) of the femoral head is the most important risk associated with the medial approach open reduction (Pospischill et al. 2012). Reportedly, re-dislocation, possible need for secondary pelvic osteotomy (secondary to limited acetabular re-modeling), limitations of ROM, and subsequent lateralization of the femoral head are other possible complications.

Arthroscopic reduction of DDH is not a common treatment because it requires clinical experience in pediatric orthopedics and arthroscopic surgery. Previous reports have described the details of the surgical technique and the outcomes of arthroscopic treatment of DDH (Eberhardt et al. 20015, Kitano et al. 2010, Öztürk et al. 2013). Arthroscopic reduction was introduced by Gross (1977). McCarthy and MacEwen (2007) reported the outcomes of arthroscopic surgery in 3 children with DDH and reported that 1 child required secondary surgery owing to failure of arthroscopic reduction. Bulut et al. (2005) reported favorable clinical and radiographic outcomes of arthroscopically assisted surgical reduction for DDH performed in 4 patients younger than 18 months in 2005 and Öztürk et al. (2013) reported similar outcomes in 9 patients in 2013. Their technique involved an anterolateral skin incision and iliopsoas tenotomy followed by exposure of the hip joint capsule to directly create anteromedial and anterolateral arthroscopic portals. We performed arthroscopic reduction without dissecting the joint capsule. Additionally, the aforementioned study used a 4.0 mm 30° arthroscope, whereas we preferred the 2.7 mm 70° arthroscope, which offers better intra-articular visualization.

Eberhardt et al. (2012) reported outcomes of arthroscopic hip reduction using sub-adductor and anterolateral portals in 5 infants. Eberhardt et al. (2014) also published another study that evaluated 9 patients (mean age 21 months) in whom surgical intervention included arthroscopic reduction combined with open peri-acetabular osteotomy. They reported promising outcomes with a mean follow-up of 15 months, particularly in patients with type 2 or 3 hips based on the Tönnis classification. Eberhardt et al. (2015) published a detailed analysis of the obstacles preventing arthroscopic reduction along with their previous experience. However, these studies included only 15 children aged under 18 months and also included 15 children aged older than 18 months. Furthermore, pelvic osteotomy was performed concomitant with arthroscopic intervention in a few children in their case series. Therefore, the effects of pelvic osteotomy on the outcomes were excluded.

Iliopsoas tenotomy is routinely performed by many surgeons to reduce the risk of AVN; however, a limited number of studies have reported the effects of iliopsoas tenotomy. Yüksel et al. (2009) showed that only one-third of the tenotomized iliopsoas were reattached and that the remaining underwent atrophy. Eberhardt et al. (2014) also reported successful results in children who did not undergo tenotomy. Notably, in our study, tenotomy was not performed in Group A. This, however, did not affect the results observed in the groups.

In light of our results as well as outcomes of previous studies, arthroscopic surgical reduction of DDH seems a promising and effective alternative to medial approach open reduction in pre-walking children. The major advantages of this technique are lesser blood loss and that it does not require wide surgical dissection of the joint capsule.

The major limitation of our study is the retrospective evaluation of prospectively followed patient groups. However, the retrospective study design and comparison of the different techniques performed by 2 different surgeons exclude any patient selection bias. The strength of this study is that it is the first to compare an arthroscopic technique with the commonly used Ludloff technique.

In conclusion, arthroscopic surgical reduction of DDH showed promising clinical and radiographic outcomes that were similar to those observed with a medial approach open reduction in patients aged 6–18 months diagnosed with DDH.

**Table 1.** Demographic data of patients

Demographic data	Medial open reduction (n = 28)	Arthroscopic reduction (n = 26)	All patients (n = 54)
Sex (female/male)	25/3	22/4	47/7
Age (months), median (range)	11 (6–17)	12 (7–17)	11 (6–17)
Side (right/left)	10/18	10/16	20/34

**Table 2.** Perioperative data of patients. Values are median (range) unless otherwise specified

	Medial open reduction (n = 28)	Arthroscopic reduction (n = 26)	All patients (n = 54)
Preoperative data			
Tönnis classification, n			
Grade I	0	0	0
Grade II	10	10	20
Grade III	14	13	27
Grade IV	4	3	7
Acetabular index angle (°)	40 (24–49)	38 (28–44)	39 (24–49)
Operative data			
Operative time (minutes)	34 (30–40)	32 (30–40)	32 (30–40)
Estimated blood loss (mL)	35 (15–55)	9 (5–15)	18 (5–55)
Postoperative data			
Acetabular index angle (°)	26 (11–39)	27 (19–36)	26 (11–39)
Coverage ratio of femoral head (%)	80 (0–100)	80 (50–100)	80 (0–100)
